# Dynamic alterations of ctDNA associate with the therapeutic outcome in the advanced non‐small cell lung cancer patients who received sintilimab plus anlotinib regime as 1st line therapy

**DOI:** 10.1002/ctm2.1277

**Published:** 2023-05-30

**Authors:** Tianqing Chu, Jie Yuan, Jun Lu, Wei Nie, Runbo Zhong, Bo Zhang, Wei Zhang, Chunlei Shi, Jialin Qian, Yanwei Zhang, Xueyan Zhang, Jiajun Teng, Zhiqiang Gao, Yuqing Lou, Jiaqi Li, Huiping Qiang, Chao Zhang, Jin Li, Xuefeng Xia, Hua Zhong, Baohui Han

**Affiliations:** ^1^ Department of Respiratory and Critical Care Medicine, Shanghai Chest Hospital Shanghai Jiao Tong University School of Medicine Shanghai China; ^2^ Geneplus‐Shenzhen Shenzhen China; ^3^ Shanghai Institute of Thoracic Oncology, Shanghai Chest Hospital Shanghai Jiao Tong University School of Medicine Shanghai China; ^4^ Translational Medical Research Platform for Thoracic Oncology, Shanghai Chest Hospital Shanghai Jiao Tong University School of Medicine Shanghai China; ^5^ Department of Bio‐Bank, Shanghai Chest Hospital Shanghai Jiao Tong University School of Medicine Shanghai China; ^6^ Geneplus‐Beijing Beijing China

1

Dear Editor,

In our previous study (NCT03628521), the efficacy and safety of sintilimab plus anlotinib regime have been evaluated upon the patients with advanced non‐small cell lung cancer (NSCLC).^1^ Liquid biopsy plays an important role in screening responders in clinical practice.^2^, ^3^, ^4^, ^5^ Therefore, understanding the circulating tumour DNA (ctDNA) as biomarker for monitoring the therapeutic outcome potentially provides new insights into the combined regime‐based stratification. In the present study, we performed an exploratory study to screen the potential ctDNA‐based biomarker.

To further understand the underlying biomarker for stratifying the responders, we performed the ctDNA analysis via customized‐panel consists of 1021 genes^6^, ^7^, ^8^ (Geneplus, China; Table S1) at four timepoints, that was baseline (BL), before cycle 2 (C2), best response (BR), all the way to after progression disease (PD). Our data showed a ctDNA positive rate of 84.2% at BL, 60% at C2, 50% at BR, and 70% at PD (Figure 1A). The correlation analysis of blood tumour mutational burden (bTMB) and tissue tumour mutational burden (tTMB) at BL indicated that there is a favourable consistency between bTMB and tTMB, suggesting bTMB could serve as a long‐term monitoring for evaluating immunotherapy outcomes (Figure S1). Consequently, we attempted to analyse the association between bTMB and progression‐free survival (PFS)/overall survival (OS). Regrettably, results showed that bTMB was not associated with PFS/OS in the NSCLC patients who received sintilimab plus anlotinib therapy (Figure S2A–‐D). Besides, we also performed the association analysis between the brain metastasis, tumour stage or PD‐L1 expression with PFS/OS, and the results indicated that these clinical characteristics are not associated with prognosis in this cohort (Figure S3). Furthermore, our data indicated that the maximum somatic allele frequency (MSAF) corrected bTMB was also not correlated with PFS/OS (Figure S4). Therefore, we wondered that whether the MSAF could be used as biomarker for stratifying the responders who received sintilimab plus anlotinib therapy.

**FIGURE 1:**
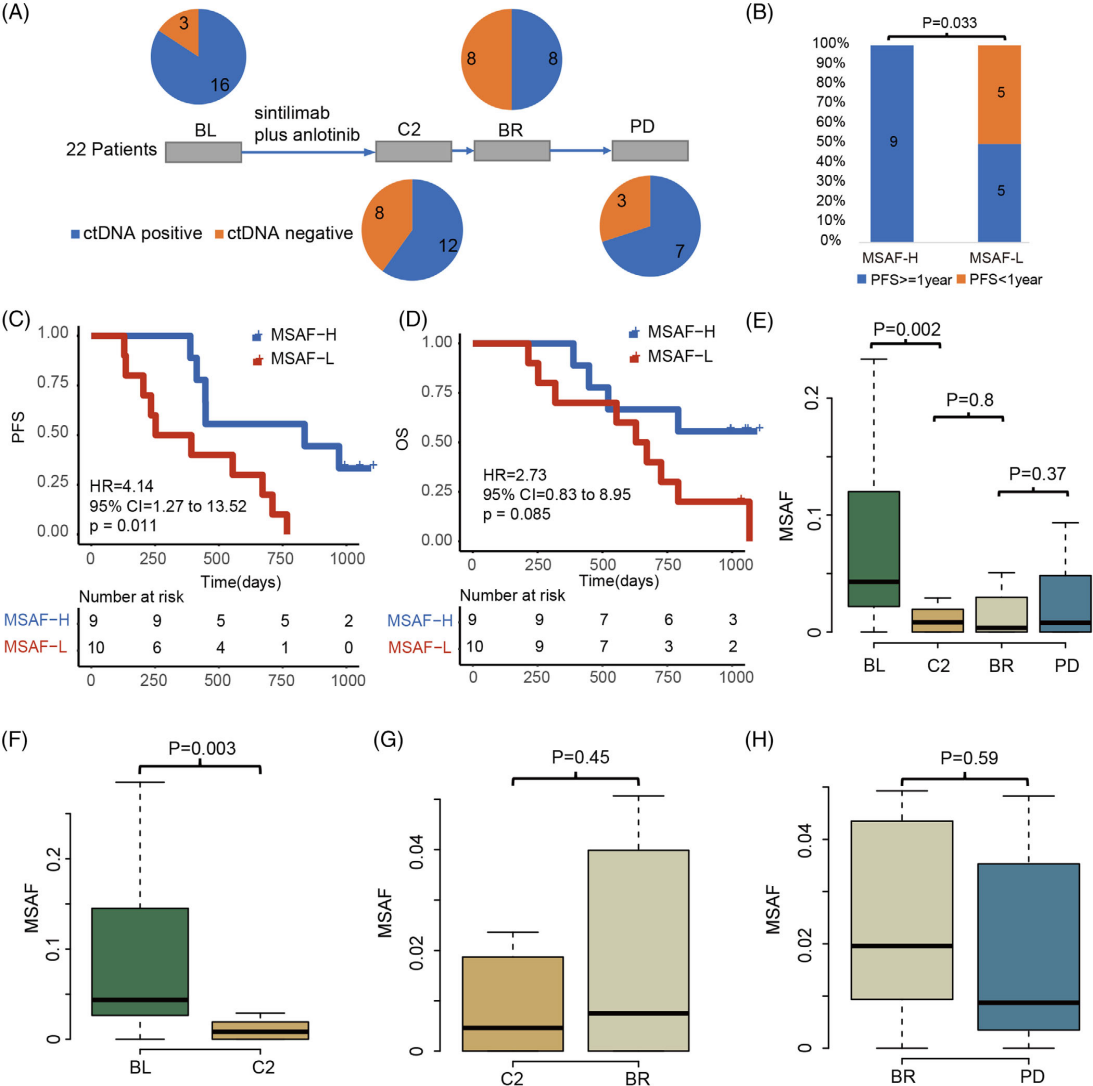
The predictive value of baseline MSAF for advanced non‐small cell lung cancer (NSCLC) patients who received sintilimab plus anlotinib as 1st line therapy. **(A)** Study diagram. Twenty‐two patients were recruited in this clinical trial. Nineteen blood samples were collected at baseline (BL), 20 blood samples were collected at C2, 16 blood samples were collected at best response (BR), and 10 blood samples were collected at progression disease (PD). The blue represents ctDNA positive, and the deep yellow represents ctDNA negative. **(B)** Progression‐free survival (PFS) enrichment for the high level of MSAF patients (MSAF‐H) and low level of MSAF patients (MSAF‐L). The blue represents the patients with PFS > 1 year, and the deep yellow represents the patients with PFS < 1 year. **(C)** Kaplan–Meier curves analysis of PFS between MSAF‐H patients and MSAF‐L patients. For MSAF‐H patients, the median PFS duration was 836 days (*n =* 9); for MSAF‐L patients, the median PFS duration was 322 days (*n =* 10). **(D)** Kaplan–Meier curves analysis of overall survival (OS) between MSAF‐H patients and MSAF‐L patients. For MSAF‐H patients, the median OS duration was 985 days (*n =* 9); for MSAF‐L patients, the median OS duration was 650 days (*n =* 10). **(E)** The difference of MSAF distribution across the different timepoints. All sequenced samples were calculated. BL: 19 samples; C2: 20 samples; BR: 16 samples; PD: 10 samples. **(F–H)** The difference of MSAF between BL and C2; C2 and BR; BR and PD. The paired samples were included for analysing the differences. **(F)** Eighteen patients have the paired samples between BL and C2. **(G)** Sixteen patients have the paired samples between C2 and BR. **(H)** Ten patients have the paired samples between BR and PD.

Interestingly, our results indicated that the NSCLC patients with high MSAF received more PFS benefit than those with low MSAF at BL (*p* = .033; Figure 1B). Furthermore, Kaplan–Meier curve analysis showed that the NSCLC patients with high MSAF received a median PFS of 836 days, while the patients with low MSAF received a median PFS of 322 days at BL (*p* = .011; Figure 1C). Regrettably, we could not obtain a similar result when the MSAF was used for OS stratification (*p* = .085; Figure 1D). These results suggested that the MSAF potentially be used as a biomarker for PFS stratification but not OS stratification.

Furthermore, we found that the MSAF levels of majority patients decreased significantly at C2 and then maintain a relative stability during the later therapy in this clinical trial (Figure 1E–H, Figure S5A–C). Therefore, we would like to know that whether the MSAF alterations from BL to C2 can be used as a valuable biomarker for PFS/OS stratification. Firstly, we defined the MSAF alterations from BL to C2 as δMSAF, which calculated via the MSAF of BL minus the MSAF of C2, then divided by MSAF of BL. Secondly, we performed Cox regression to investigate the association between MSAF/δMSAF and PFS/OS. The results indicated that the hazard ratios derived from δMSAF stratification show better performance than those of MSAF (Figure 2A–F). Lastly, we performed PFS/OS analysis via δMSAF‐based stratification. Results suggested that the NSCLC patients with high δMSAF received more PFS benefit than those with low δMSAF the patients (*p* = .029; Figure 2G). Furthermore, Kaplan–Meier curve analysis showed that the NSCLC patients with high δMSAF received more PFS and OS benefit than those patients with low δMSAF (PFS: *p* = .027, Figure 2H; OS: *p* = .032, Figure 2I).

**FIGURE 2:**
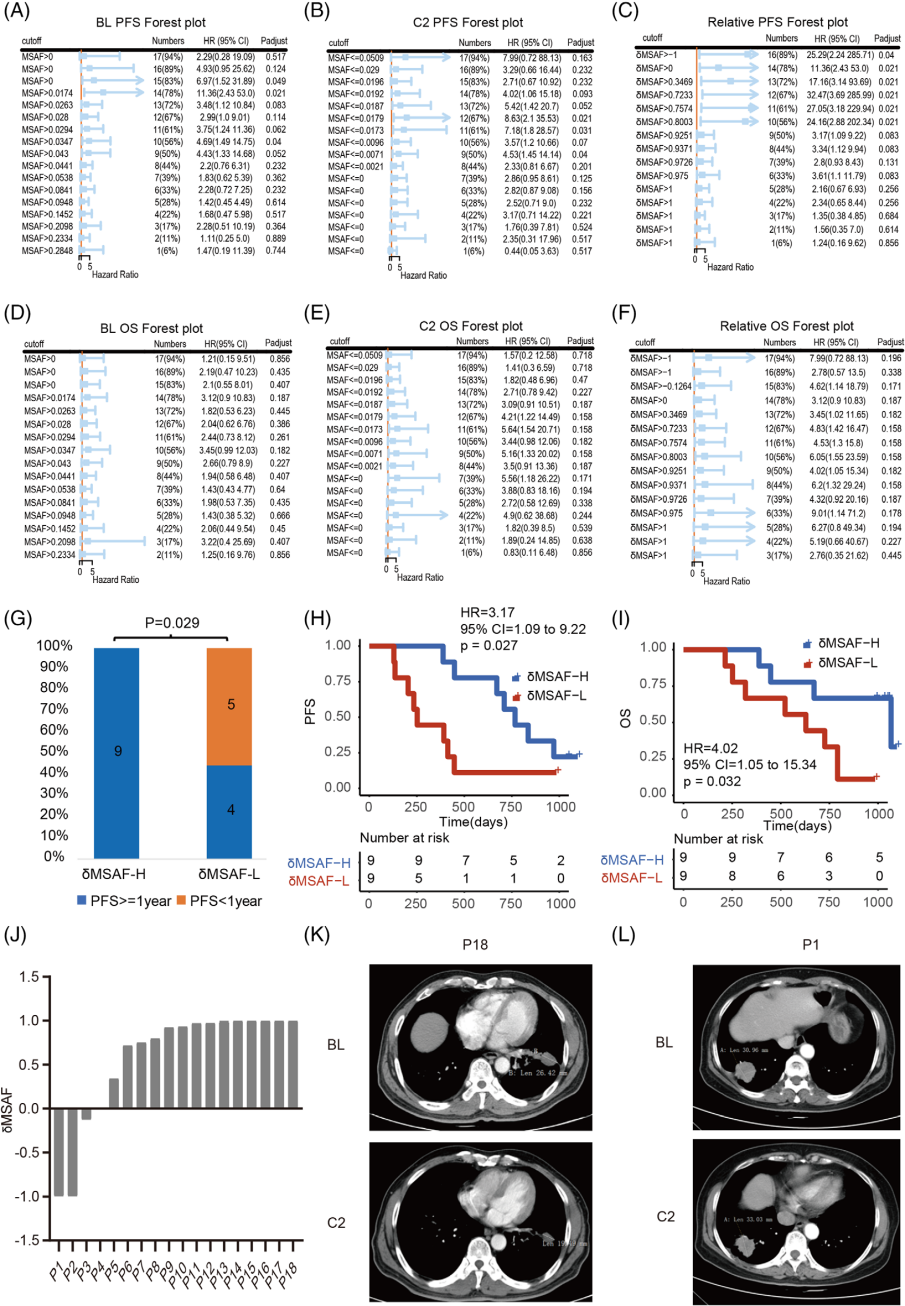
The predictive value of δMSAF for advanced non‐small cell lung cancer (NSCLC) patients who received sintilimab plus anlotinib as 1st line therapy. **(A–C)** Cox regression of the association between MSAF/δMSAF and progression‐free survival (PFS) by serially shift the cut‐off. **(A)** The MSAF at baseline (BL), **(B)** the MSAF at C2, and **(C)** the δMSAF. **(D–F)** Cox regression of the association between MSAF/δMSAF and overall survival (OS) by serially shift the cut‐off. **(D)** The MSAF at BL, **(E)** the MSAF at C2, and **(F)** the δMSAF. **(G)** PFS enrichment for the high level of δMSAF patients (δMSAF‐H) and low level of δMSAF patients (δMSAF‐L). The blue represents the patients with PFS > 1 year, and the deep yellow represents the patients with PFS < 1 year. **(H and I)** Kaplan–Meier curve analysis of PFS and OS between δMSAF‐H patients and δMSAF‐L patients. For δMSAF‐H patients, the median PFS and OS duration were 767 days and 1027 days, respectively (*n =* 9); for δMSAF‐L patients, the median PFS and OS duration was 252 days and 629 days respectively (*n =* 9). **(J)** The histogram showed the δMSAF for each patient (*n* = 18). **(K and L)** Representative computed tomography (CT) images of the NSCLC patients between BL and C2. **(K)** The CT images were derived from P18 (patient 18), and **(L)** the CT images were derived from P1 (patient 1).

To further understand the association between δMSAF and tumour volume, we performed the analysis of the images of computed tomography (CT) as well as the δMSAF for each patient. Among the 18 patients who have δMSAF, the δMSAF values of majority patients are greater than zero (Figure 2J). The results of combined analysis of CT images and δMSAF indicated that the greater the tumour volume shrinked, the greater the δMSAF obtained, suggesting the more potential predictive value of the δMSAF for responsive stratification of the NSCLC patients who received sintilimab plus anlotinib as 1st line therapy (Figure 2K,L, Figures S6 and S7). Lastly, we evaluated the predictive value of MSAF, which derived from C2 and BR. Results suggested that the MSAF both from the C2 and BR also have predictive value (Figure S8).

Based on the abovementioned results, we offered the first evidence that the dynamic alterations of ctDNA associate with the therapeutic outcome in the advanced NSCLC patients who received sintilimab plus anlotinib regime as 1st line therapy. Previously, we reported that the MSAF at BL potentially guided PFS stratification for the advanced NSCLC patients who received anlotinib as 3rd or more line therapy.^9^ Consistence with previous results, here, we also found that the MSAF at BL potentially guided PFS stratification for the advanced NSCLC patients who received sintilimab plus anlotinib as 1st line therapy. However, the MSAF at BL could not guide the OS stratification. Interestingly, here, we found the δMSAF not only has predictive value for guiding PFS stratification but also has predictive value for guiding OS stratification. Therefore, the δMSAF provided the potential value for clinical practice.

The limitations of this study were summarized. Firstly, the present cohort consisted a limited sample size due to the biomarker screening based on the phase I clinical trial (NCT03628521). However, the phase II clinical trial (NCT04124731) consisted of 89 patients has been finished during November 2019 to July 2022. The corresponding validation will be performed in the extensive cohort, and the validated results will be opened in future. Secondly, the present study found the high MSAF associated with better PFS; however, the previous studies showed the low MSAF associated with better PFS.^10^ For understanding the phenomenon, the association between blood TMB and MSAF was interrogated, and the results demonstrated that MSAF and bTMB were positively correlated (Figure S9). This result provided evidence that MSAF could be used as the potential biomarker for the regime of sintilimab plus anlotinib. In any event, the contradictory results provided a novelty thought for developing the predictive biomarker‐δMSAF.

Collectively, this study provided a candidate biomarker‐δMSAF that potentially be used for stratifying the responders for the NSCLC patients who received sintilimab and anlotinib as the 1st line therapy and offered a novelty perspective for screening liquid biopsy‐based biomarker via ctDNA sequencing.

## CONFLICT OF INTERST STATEMENT

JY, CZ, JL and XX are current employees of Geneplus Company. No other actual or potential conflict of interest is declared.

## Supporting information

Supporting Information

Supporting Information
