# Supplementary material for: Dynamic alterations of ctDNA associate with the therapeutic outcome in the advanced non‐small cell lung cancer patients who received sintilimab plus anlotinib regime as 1st line therapy
Source: Clin Transl Med. 2023 May 30;13(6):e1277. doi: 10.1002/ctm2.1277 (PMC10230154; doi:10.1002/ctm2.1277)
Supplement: Supplementary file 1 — Supporting Information [file CTM2-13-e1277-s002.docx]

**Supplementary Figures**


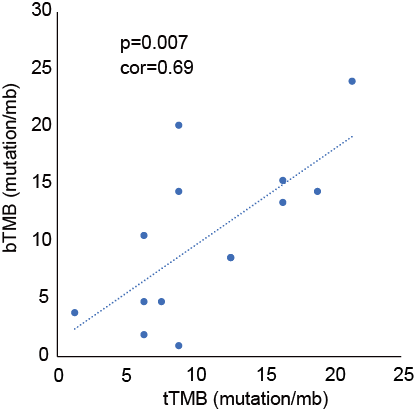


**Supplementary Figure 1, The correlation between blood tumor mutational burden and tissue tumor mutational burden at baseline.**


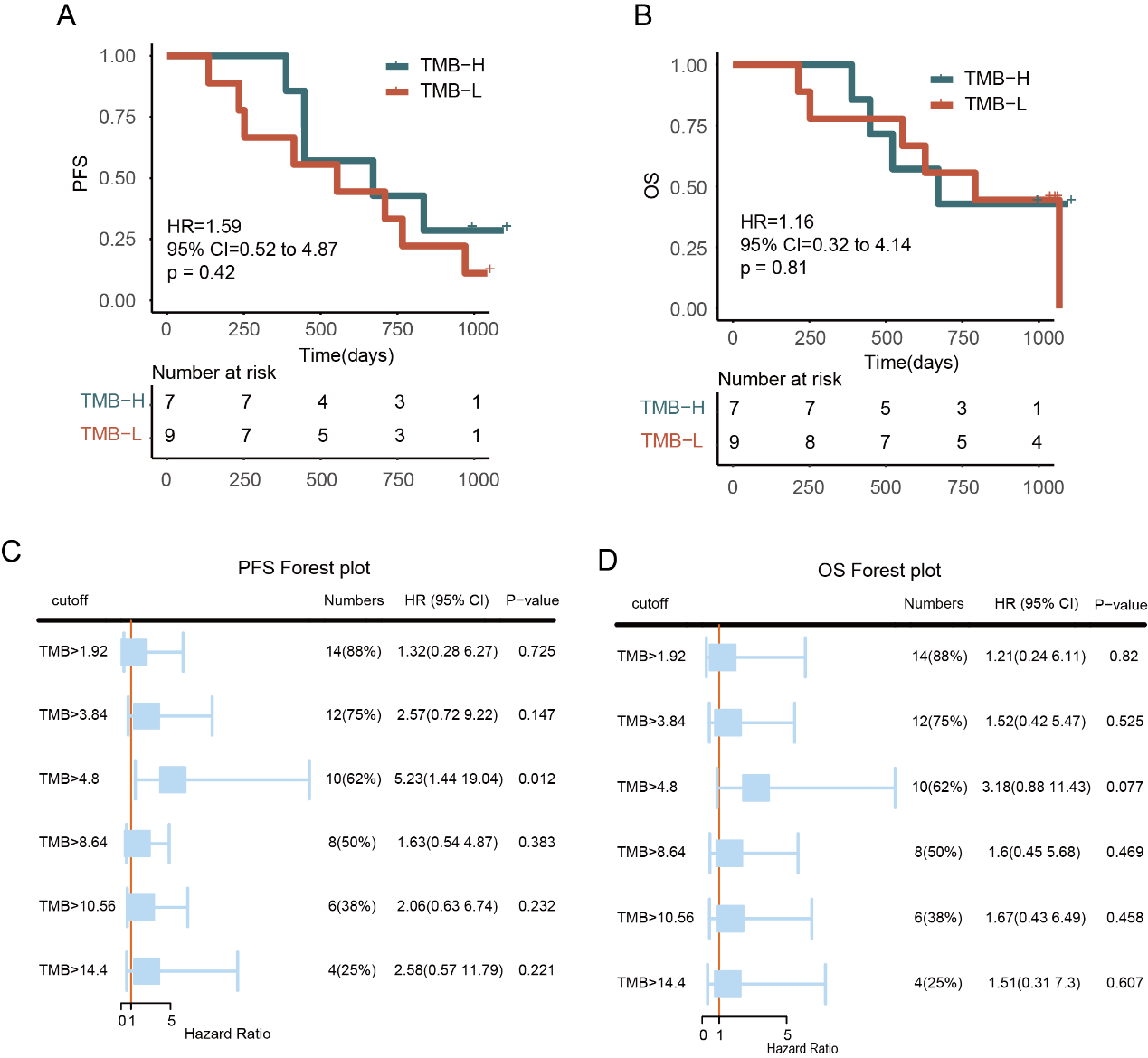


**Supplementary Figure 2, Association of survival benefits with the bTMB at baseline. (A-B)** Kaplan-Meier survival curves of **(A)** PFS and **(B)** OS between bTMB-H and bTMB-L at baseline; **(C-D)** Cox regression of the association between bTMB and **(C)** PFS, **(D)** OS by serially shift the cut-off at baseline.

**
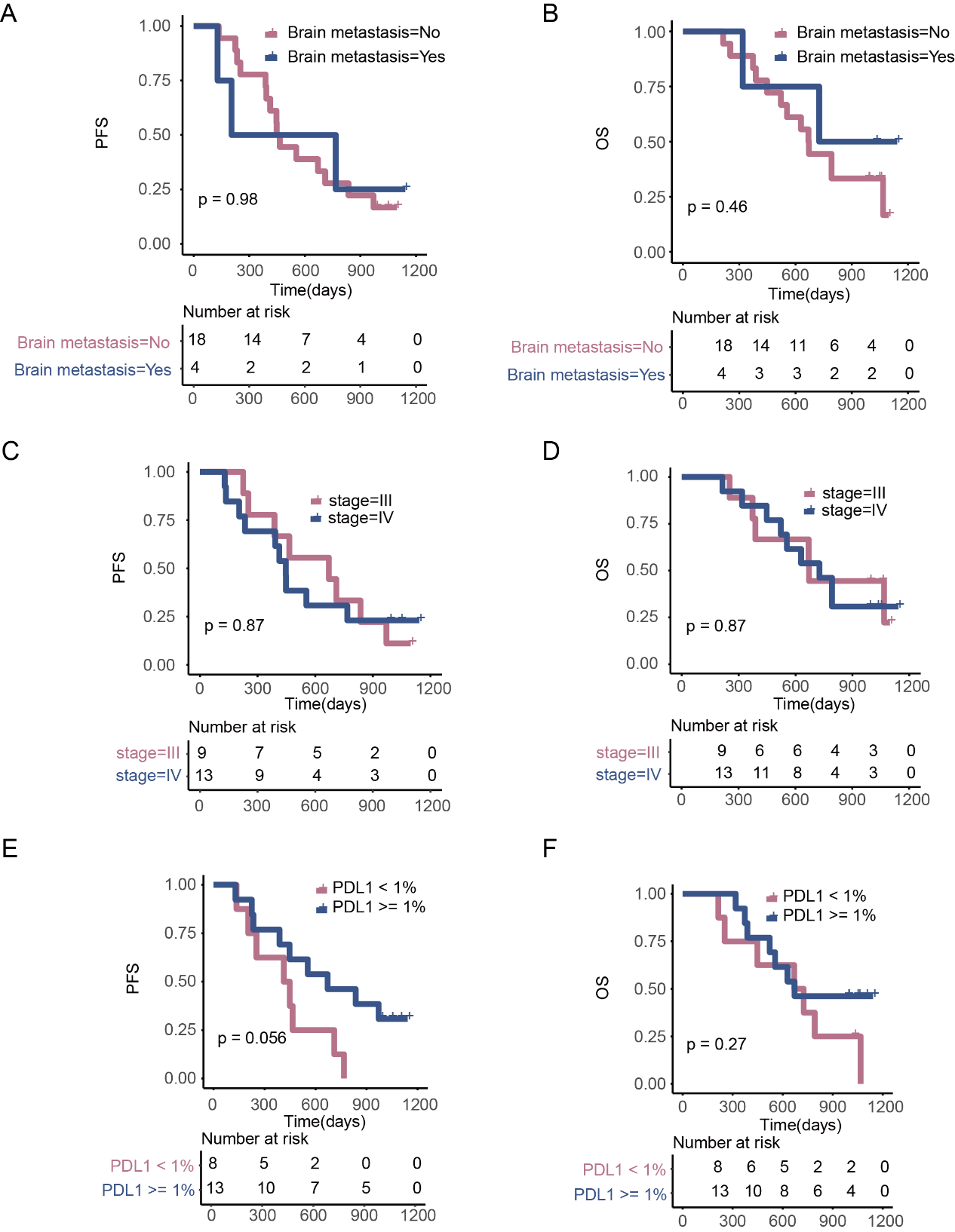
**

**Supplementary Figure 3, Association of survival benefits with the brain metastasis, cancer Stage and PD-L1 expression. (A-B) Kaplan-Meier survival curves of (A) PFS and (B) OS between patients with and without brain metastasis. (C-D) Kaplan-Meier survival curves of (C) PFS and (D) OS between stage III and stage IV patients. (E-F) Kaplan-Meier survival curves of (E) PFS and (F) OS between patients of PDL1 expression < 1% and PDL1 expression >= 1%.**


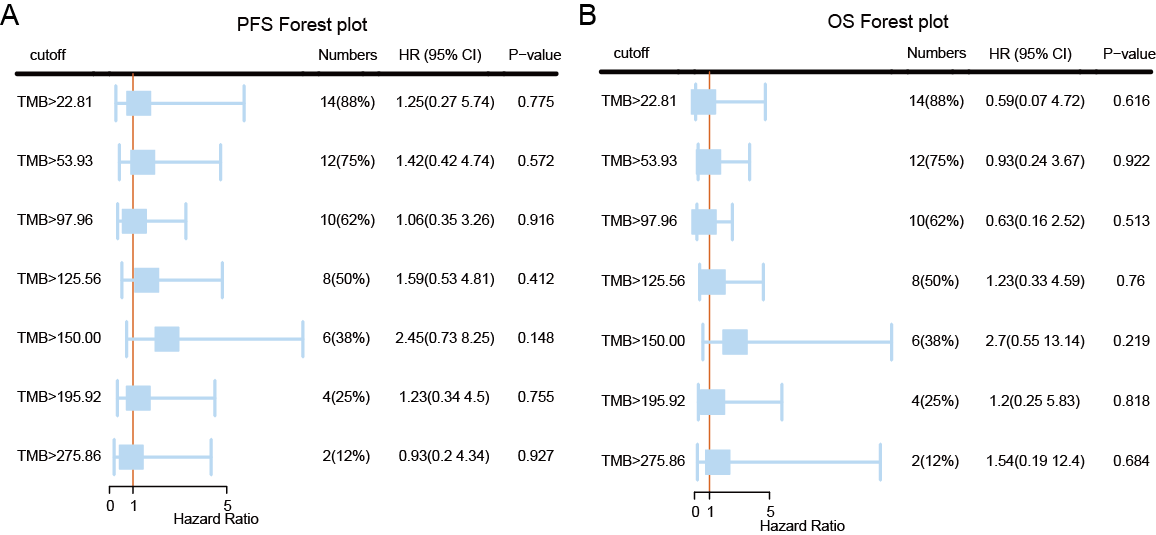


**Supplementary Figure 4, Cox regression of the association between MSAF corrected bTMB and (A) PFS, (B) OS by serially shift the cut-off at baseline.**


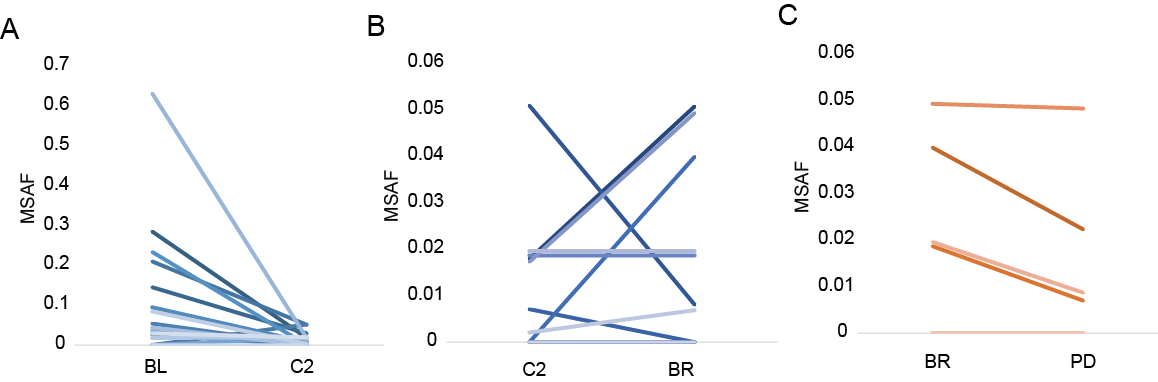


**Supplementary Figure 5, Line chart of the dynamic alterations of MSAF between two neighboring timepoints. (A) BL and C2; (B) C2 and BR; (C) BR and PD.**


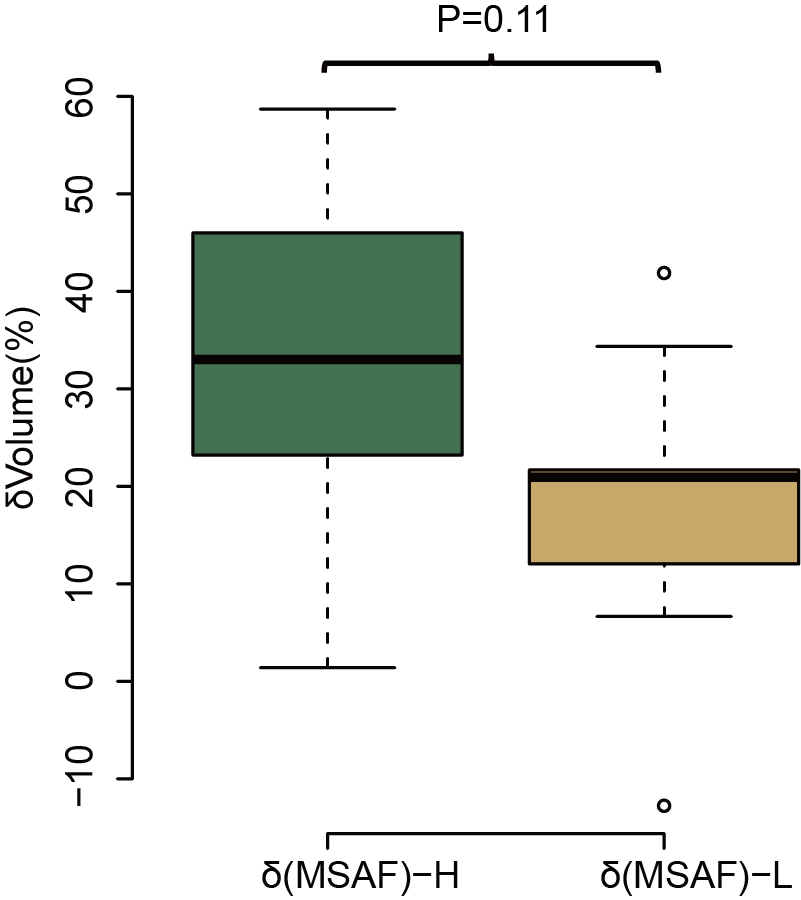


**Supplementary Figure 6, The association of δMSAF and the proportion of tumor volume reduction from BL to C2.**

**
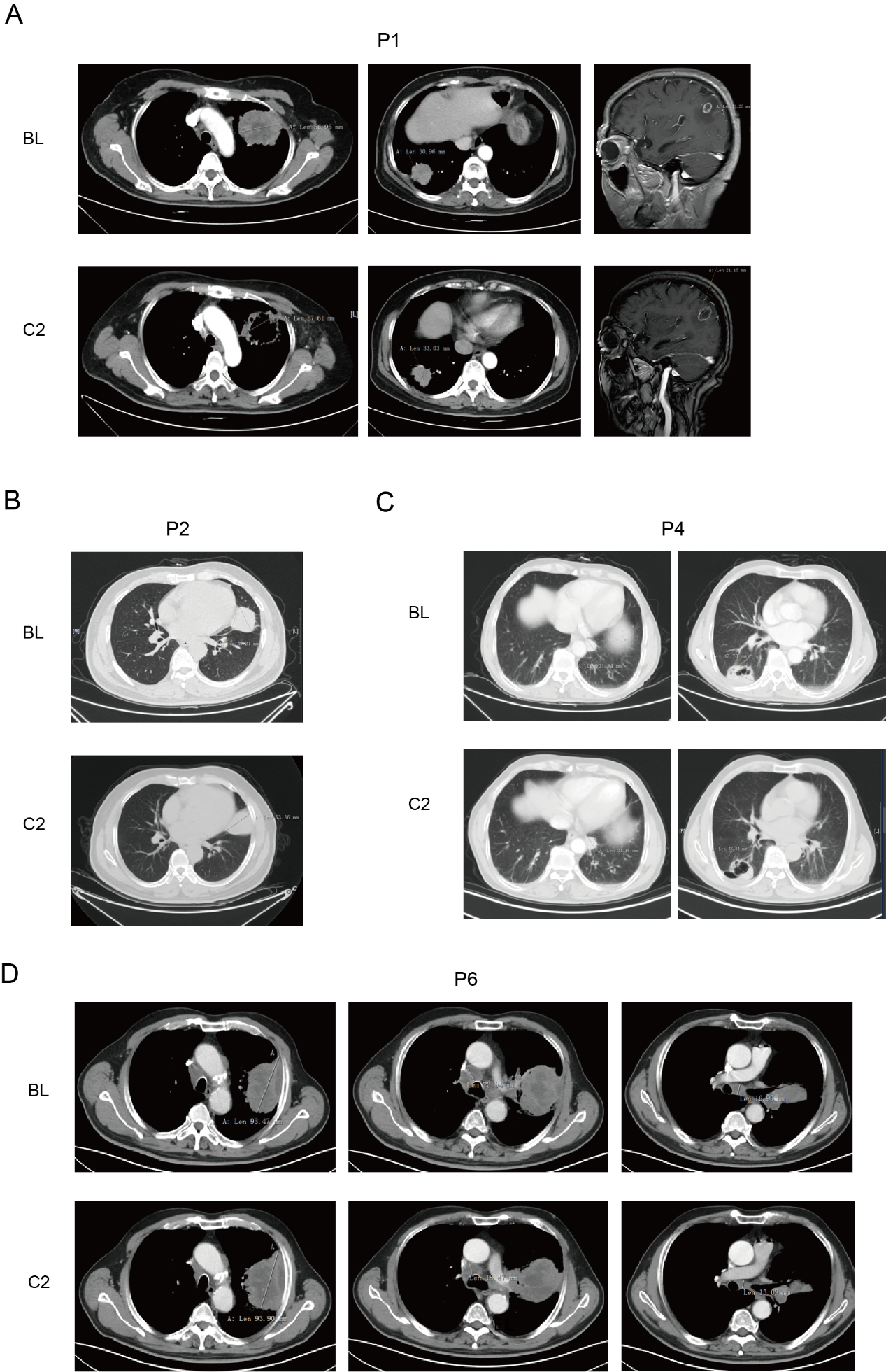
**

**
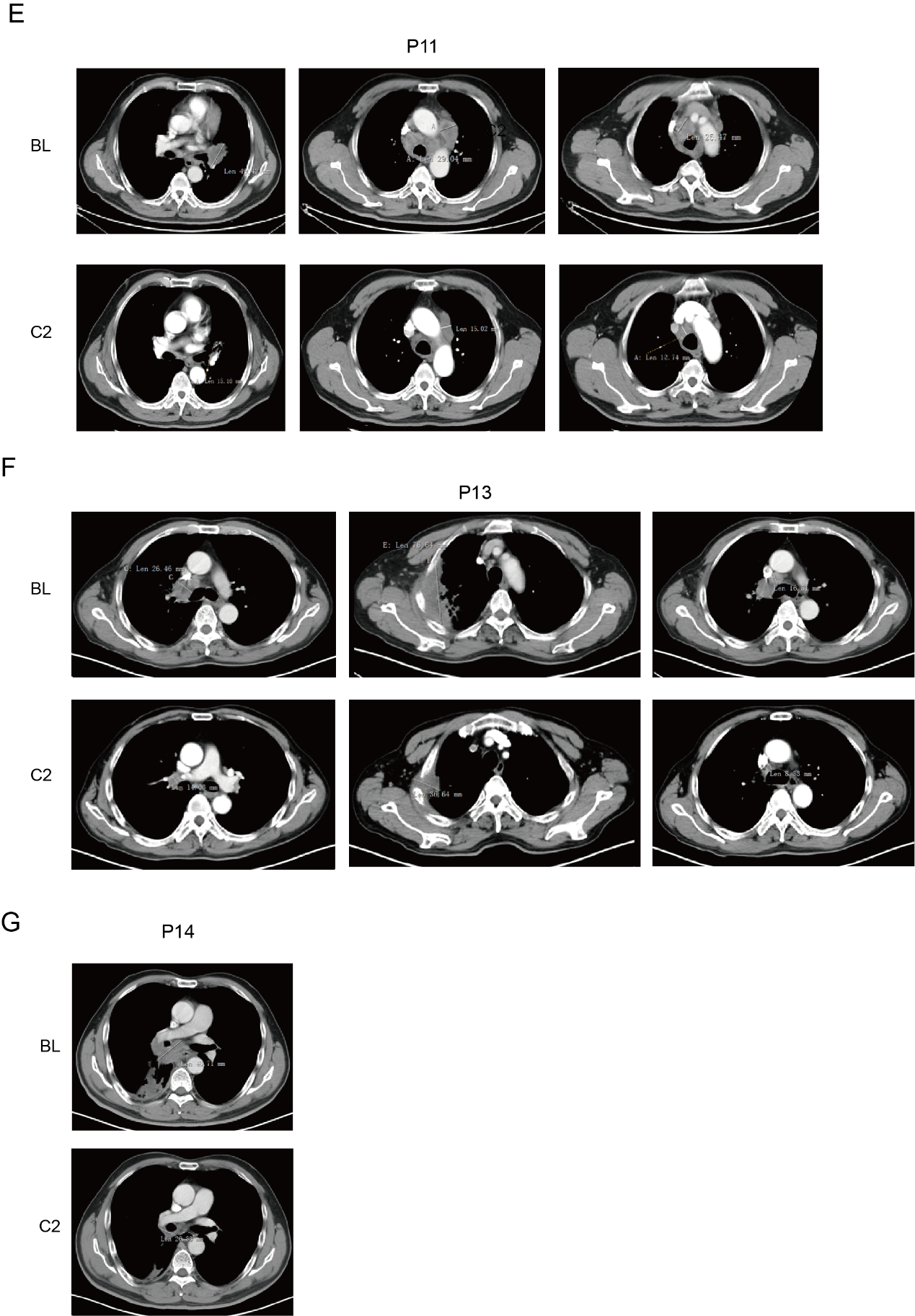
**

**
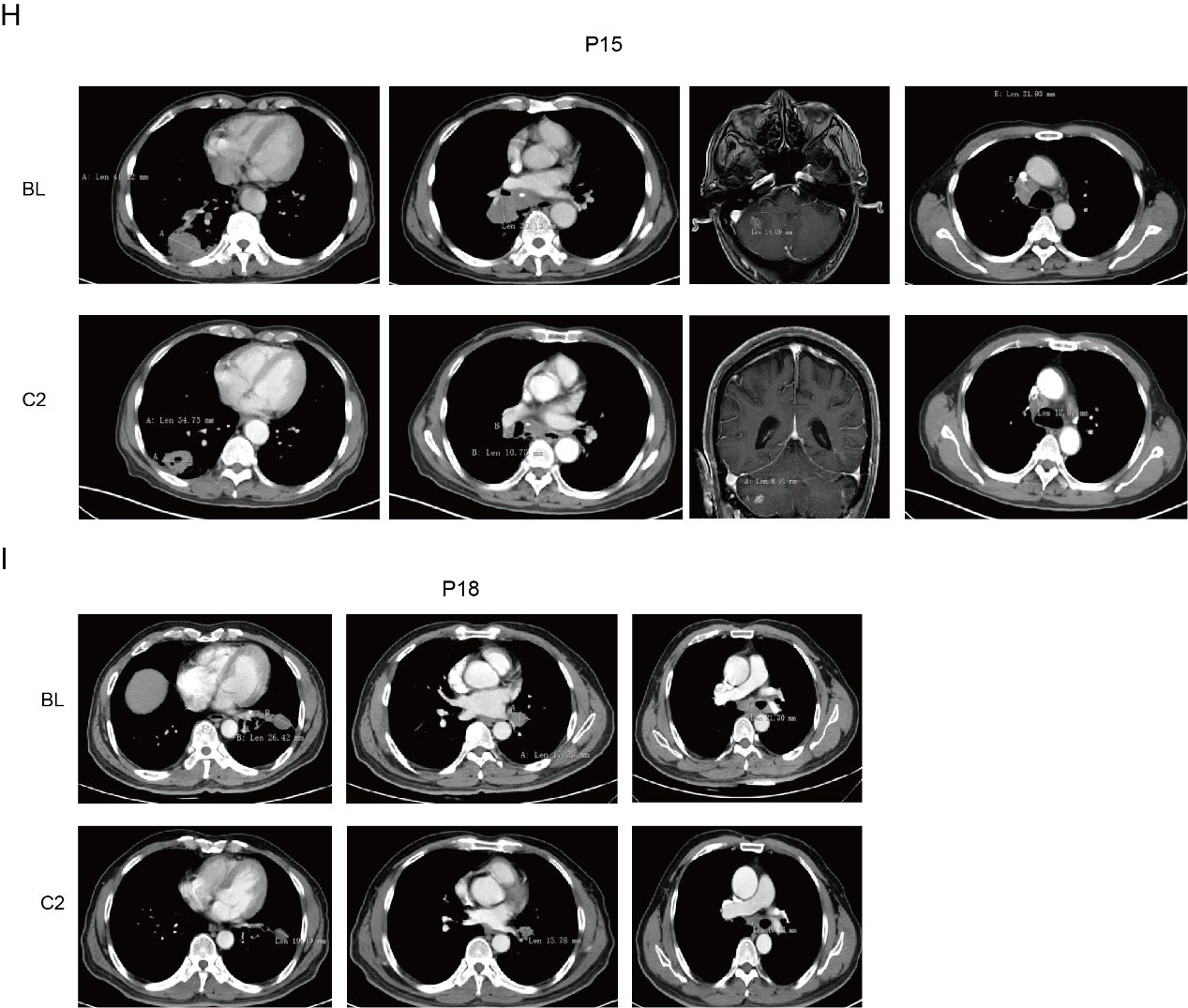
**

**Supplementary Figure 7, Representative CT images of the NSCLC patients between BL and C2. (A-I) The CT images were derived from patient 1(A), patient 2(B), patient 4(C), patient 6(D), patient 11(E), patient 13(F), patient 14(G), patient 15(H), patient 18(I).**


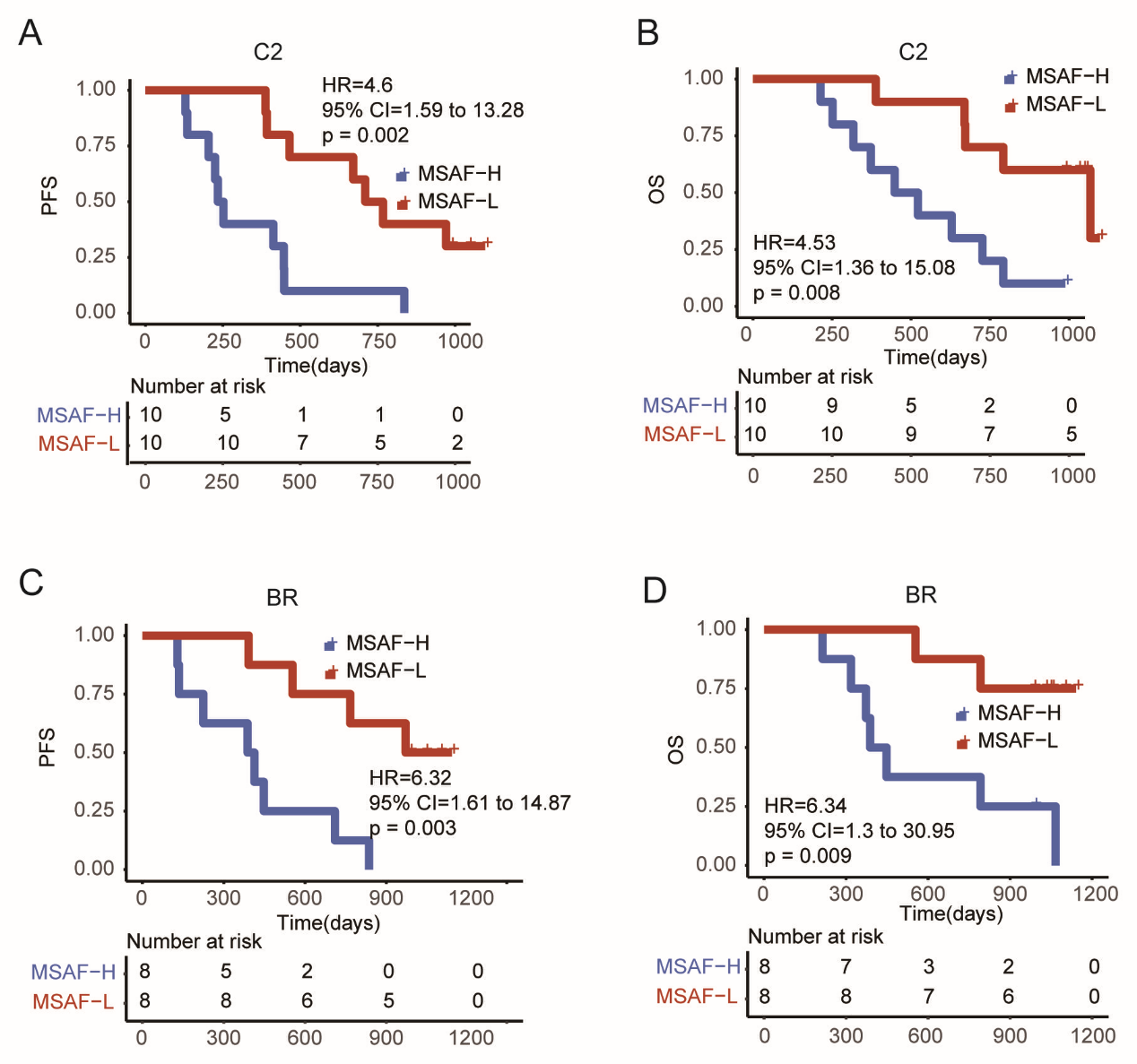


**Supplementary Figure 8, Association of survival benefits with the MSAF from single post-treatment timepoints. (A-B) Kaplan-Meier survival curves of (A) PFS and (B) OS between MSAF-H and MSAF-L patients in C2 (median MSAF as the cut-off). (C-D) Kaplan-Meier survival curves of (C) PFS and (D) OS between MSAF-H and MSAF-L patients in BR (median MSAF as the cut-off).**


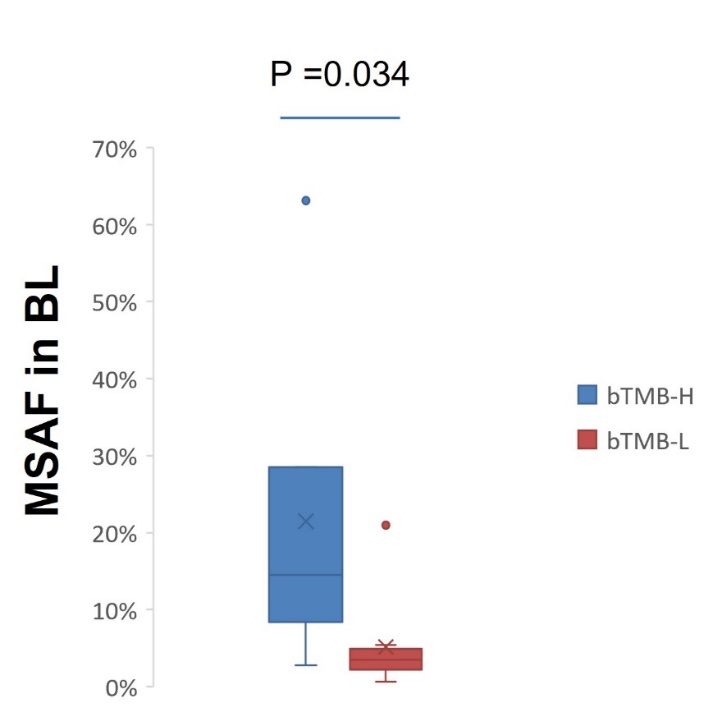


**Supplementary Figure 9, The association of b-TMB and MSAF in BL.**

**Supplementary Methods**

A total of 22 advanced NSCLC patients who received sintilimab plus anlotinib as 1^st^ line therapy were recruited in this clinical trial (NCT03628521). This clinical trial was conducted in accordance with the Declaration of Helsinki and Good Clinical Practice guidelines. This study was reviewed and approved by the institutional review board and ethics committee at Shanghai Chest Hospital. All patients provided written informed consent before enrollment. The inclusion criteria for eligible patients and the therapeutic details were consistent with our previous report^1^. Clinical endpoints included PFS and OS were evaluated according to the standard of RECIST1.1, and adverse events (AEs) were evaluated according to the U.S. National Cancer Institute’s Common Terminology Criteria for Adverse Events (version 4.0). The median follow-up time for this clinical trial was 670 days and the last follow-up date was 6^th^, November 2021.

The blood samples for each patient from multiple timepoints [baseline (BL), before cycle 2 (C2), best response (BR), all the way to after progression disease (PD)] were collected, and then performed ctDNA sequence via customized-panel consists of 1021 genes (Geneplus, China). Totally, 65 blood samples were performed ctDNA analysis (Figure 1A). Of the 65 blood samples, 19 blood samples were collected at BL, 20 blood samples were collected at C2, 16 blood samples were collected at BR, and 10 blood samples were collected at PD. Barcoded libraries were hybridized to the abovementioned customized panel containing whole exons, selected introns of 288 genes, and selected regions of 733 genes for ctDNA sample (Supplementary Table 1). Detailed gene list was described in the previous study^2^. The somatic mutations in each ctDNA sample were called with in-house criteria^3^. The MSAF was defined as the maximum allele frequency (AF) of all the somatic mutations identified per sample by ctDNA sequencing. The δMSAF was defined as the MSAF of BL minus the MSAF of cycle 2 (C2), then divided by MSAF of BL. Here, based on the paired samples between BL and C2, 18 patients have the δMSAF. The median MSAF/δMSAF was set as the cutoff for classifying high level or low level.

The tissue TMB (tTMB) was measured by the FoundationOne CDx assay (Foundation Medicine, Cambridge, MA)^4,5^. The blood TMB (bTMB) was measured by Geneplus 1021 genes panel. TMB was calculated as the number of somatic synonymous and nonsynonymous SNVs and small indels in exon regions divided by the length of the coding region for 1021 genes (1.04Mb). Based on the 75% percentile of the bTMB of Geneplus NSCLC blood samples (n = 4243)6, the cut-off for TMB-H and TMB-L was set as 8.64 mutations/Mb.

Survival analysis was performed with Kaplan-Meier curves, and the *P* value was determined by log-rank test. Enrichment analysis between two groups were performed by the Mann-Whitney test. Cox regression was used to study the association between MSAF and survival benefit, with hazard ratio (HR) and their 95% confidence intervals (CIs). All reported *P* values were two-tailed, and *P* < 0.05 was considered significant. All data analyses were performed using R (version 3.6.1) and GraphPad Prism (version 8.0.2).

**References**

1 Chu, T. *et al.* Phase 1b Study of Sintilimab Plus Anlotinib as First-line Therapy in Patients With Advanced NSCLC. *Journal of Thoracic Oncology* **16**, 643-652, doi:<https://doi.org/10.1016/j.jtho.2020.11.026> (2021).

2 Wang, D. *et al.* Preoperative evaluation of microvascular invasion with circulating tumour DNA in operable hepatocellular carcinoma. *Liver Int* **40**, 1997-2007, doi:10.1111/liv.14463 (2020).

3 Zhou, J. *et al.* Serial Circulating Tumor DNA in Predicting and Monitoring the Effect of Neoadjuvant Chemoradiotherapy in Patients with Rectal Cancer: A Prospective Multicenter Study. *Clin Cancer Res* **27**, 301-310, doi:10.1158/1078-0432.CCR-20-2299 (2021).

4 Chalmers, Z. R. *et al.* Analysis of 100,000 human cancer genomes reveals the landscape of tumor mutational burden. *Genome Med* **9**, 34, doi:10.1186/s13073-017-0424-2 (2017).

5 Goodman, A. M. *et al.* Tumor Mutational Burden as an Independent Predictor of Response to Immunotherapy in Diverse Cancers. *Mol Cancer Ther* **16**, 2598-2608, doi:10.1158/1535-7163.MCT-17-0386 (2017).

6 Zhang, Y. *et al.* Pan-cancer circulating tumor DNA detection in over 10,000 Chinese patients. *Nat Commun* **12**, 11, doi:10.1038/s41467-020-20162-8 (2021).
